# *Neospora caninum* and/or *Toxoplasma gondii* Seroprevalence: Vaccination against PCV2 and Muscle Enzyme Activity in Seropositive and Seronegative Pigs

**DOI:** 10.3390/microorganisms9051097

**Published:** 2021-05-20

**Authors:** Labrini V. Athanasiou, Vasileios G. Papatsiros, Victoria M. Spanou, Eleni G. Katsogiannou, Anna Dedousi

**Affiliations:** 1 Department of Medicine, Faculty of Veterinary Medicine, University of Thessaly, 43100 Karditsa, Greece; vpapatsiros@vet.uth.gr (V.G.P.); vispanou@uth.gr (V.M.S.); elkatsog@uth.gr (E.G.K.); 2 Veterinary Research Institute, HAO-Demeter, 57001 Thessaloniki, Greece; dedousi@vri.gr

**Keywords:** aspartate aminotransferase, creatine kinase, immunosuppression, *Neospora caninum*, PCV2, pigs, serology, *Toxoplasma gondii*, vaccination

## Abstract

*Neospora caninum* and *Toxoplasma gondii* affect both humans and animals worldwide. To investigate their seroprevalence and differences in seropositivity between pigs vaccinated and unvaccinated against porcine circovirus 2 (PCV2), as well as differences in muscle enzyme activity between seropositive and seronegative pigs, blood samples were collected from 380 sows. Antibodies against *T. gondii* and *N. caninum* were detected by an indirect immunofluorescence antibody (IFA) assay, while the activities of creatine kinase (CK) and aspartate aminotransferase (AST) were biochemically assessed. Out of the 364 sows finally included in the study, 4.4%, 3.5%, and 0.5% were seropositive to *T. gondii*, *N. caninum*, or both. A significantly higher percentage of seropositivity against *T. gondii* and/or *N. caninum* in PCV2 unvaccinated pigs compared with vaccinated pigs was observed. Increased serum activities of CK and AST were detected in 71.43% and 100% of only against *T. gondii* (T+) and 63.64% and 90.91% of only against *N. caninum* (N+) seropositive sows, respectively, and were significantly higher compared to seronegative animals. *T. gondii* and *N. caninum* seropositivity, especially in presumed immunocompromised pigs, and the evidence of muscle damage highlight their importance as a zoonotic pathogen and animal model of human infection, respectively.

## 1. Introduction

Protozoan parasites of the phylum Apicomplexa infect several animal species, while some of them are of zoonotic importance. *Toxoplasma gondii* and *Neospora caninum* are closely related intracellular protozoan parasites belonging to the Coccidia subclass [1]. Tissue cyst formation and transmission via environmentally shed sporulated oocysts are some of their shared biological features [2,3]. Both organisms follow an indirect transmission cycle with carnivores being the definitive hosts and a broad spectrum of mammalian species, including humans and birds, serving as intermediate hosts. The main routes of infection in both humans and animals is by the ingestion of coccidian oocysts spread in the environment by infected carnivores’ feces or after consumption of encysted bradyzoites of the intermediate hosts’ tissues [3].

*T. gondii* is zoonotic, infecting 30–50% of the world’s human population [4]. Over 1 million cases of toxoplasmosis are estimated every year in Europe [5]. *N. caninum* is unlikely to cause disease in immunocompetent individuals. However, although the presence of the parasite as well as antibodies against *N. caninum* have been reported in humans [6], the zoonotic potential of neosporosis needs further elucidation. Research on the prevalence and epidemiology of *T. gondii* among different countries and animal species is critical for the development of prevention strategies for human infection. In a meta-analysis study with data from 47 countries, the pooled global seroprevalence in swine populations was 19%, with Europe (13%; 10–15%) presenting the lowest and Africa (25%; 17–34%) and North America (25%; 19–33%) the highest seroprevalence [7]. This high prevalence indicates that pigs are an important source of *T. gondii* worldwide, while the consumption of pork contaminated with tissue cysts has a huge impact on the transmission to humans [8].

Recently, serological studies have reported the occurrence of *N. caninum* in breeding pigs worldwide. The first *N. caninum* natural infection was evidenced in Germany in 2004 with only one seropositive pig, western blot confirmed, out of the 2041 animals tested [9]. Most studies refer to the different states of Brazil with seroprevalence presenting an ascending trend over the years from 3.1% in 2010 [10] and 3.2% in 2014 [11], to 13.49% in 2019 [12]. In addition, a seroprevalence of 1.9% has been reported in 2019 for the first time in pigs in China, with a range of 0.3–4.6% in the different regions [13], while in the Czech Republic, 3% of swine were seropositive, with 1.5% presenting antibodies against both *T. gondii* and *N. caninum* [14]. Referring to wildlife, 15.8% of feral swine populations in the United States were found seropositive against *N. caninum* [15]. To date, there is a paucity of data referring to swine exposure to *N. caninum* in Greece.

In Greece, the seroprevalence of *N. caninum* varies among different species. In dogs the seroprevalence was 7.63% [16], in sheep 2.5–16.8% [17,18], and in goats 6.9% [18], while in cattle population, the highest seroprevalence of 20.89–21.03% has been reported [19,20]. In wild animals, the seroprevalence seems to be low, with 1.1% in wild boars [21] and 0.95% in hares [22]. Moreover, specific antibodies against *T. gondii* were detected in different studies in Greece, with seroprevalence varying from 24.1–37% in humans [23], 48.6–56.3% in sheep [17,18,24], 30.7–61.3% in goats [18,24], 8.13% in cows [20], 4.3% in swine [25], and 1.8% in horses [26]. Regarding wildlife, the seroprevalence was 5.7% in brown hares [22] and 5.2% in the wild boar population [21].

Immunocompromised hosts are more susceptible to develop severe infection with both opportunistic pathogens *T. gondii* and *N. caninum,* while immunocompetent individuals remain mostly asymptomatic [27,28]. Polymyositis and myocarditis as a result of *T. gondii* infection has been described in immunocompetent humans [29,30,31] and dogs [32], with elevated serum values of aspartate aminotransferase (AST) and creatine kinase (CK). Referring to cats, the definitive host of the parasite, chronic and diffuse inflammatory myopathy associated with elevated CK activity and a history of intermittent lameness has been reported in a seropositive against *T. gondii* adult cats [33]. Cardiac and skeletal muscle involvement has been observed in aborted fetuses or congenitally infected young pigs [34]. Moreover, among the histopathological findings in piglets with clinical toxoplasmosis were the granulomatous myositis of skeletal muscles and necrosis of myocardial cells [35].

Clinical signs like apathy and hypothermia, as well as hematological and biochemical alterations including leukocytosis due to lymphocytosis and increased serum levels of AST and normal gamma glutamyl-transferase (GGT) have been recently reported in an experimental study in sows [36]. This was the first study evaluating the clinical signs and laboratory findings of Neosporosis in swine. However, the effects of *N. caninum* natural infection on the neuromuscular system in this species remains unclear.

The aims of this study were (a) to detect the presence of antibodies against *T. gondii* and/or *N. caninum* in pigs, (b) to identify differences in seropositivity against *T. gondii* and/or *N. caninum* between pigs vaccinated and unvaccinated against porcine circovirus 2 (PCV2), and c) to identify differences in muscle enzyme activity between seropositive and seronegative to *T. gondii* and/or *N. caninum* pigs.

## 2. Materials and Methods

### 2.1. Ethical Approval

All procedures were done according to the ethical standards in the Helsinki Declaration of 1975, as revised in 2000, as well as the national law and after receiving approval (Animal Use Ethics Committee of Veterinary Faculty University of Thessaly approval code: 65/26-02-2019 and approval date: February 2019) from our Institutional Animal Use Ethics Committee.

### 2.2. Inclusion Criteria

#### 2.2.1. Farms

A total of 31 pig farms on the Greek mainland were included in the study (Table 1). The selected 31 farms had totally a population of around 11,500 sows, which represents approximately 20% of the entire capacity of Greek swine production. Moreover, sampling was performed after receiving the farmer’s informed consent, followed by filling in a questionnaire on the herd vaccination scheme. Inclusion criteria of farms were as follows:

- Minimum capacity of 50 sows.
- Operation type of exclusive farrow-to-finish farms.
- Vaccination of gilts/sows against Aujeszky’s disease virus, parvovirus, atrophic rhinitis, erysipelas, Porcine Reproductive and Respiratory Virus (PRRSV), *Escherichia coli*, and *Clostridium* infections. Vaccination of weaners against *Mycoplasma hyopneumoniae*.
- Regular control of endo/ectoparasites by administration of ivermectin in sows.
- Single housing systems of sows with farrowing crate, including cast iron slat and solid plastic slat.
- Implementation of environmental conditions and stocking density in compliance with swine welfare requirements (Council Directive 2008/120/EC of 18 December 2008).
- Implementation of good biosecurity practices to reduce the likelihood of disease introduction and/or spread.
- Balanced diet (essential amino acids, minerals, and vitamins) according to National Research Council [37].
- Regular use of toxin binders in the feed of sows during gestation and lactation period and in the feed of weaning pigs.

#### 2.2.2. Animals

Clinically healthy sows of parity 1 and 2 at the 10th day of lactation were selected for the study. Sows with prior injection either for drug administration or blood collection within the last 7 days were excluded.

### 2.3. Sampling

Blood samples were obtained from 380 sows via puncture of the vena jugular externa with a 19-gauge needle into one vacutainer (Venoject, Terumo Europe, Leuven, Belgium) with no anticoagulant for serum retrieval. Samples were transferred to the Diagnostic Laboratory of the Faculty of Veterinary Medicine, School of Health Sciences, University of Thessaly, Greece, and placed in a cooler, with icepacks avoiding direct contact with the tubes. Within 2 h after collection, blood samples were centrifuged at 300× *g* for 10 min. Hemolyzed blood specimens were excluded from the study to avoid any possible interference in the biochemistry results. The serum was recovered, transferred into three plastic vials (Eppendorf Tubes, Eppendorf AG, Hamburg, Germany), and frozen immediately at −20 °C pending analysis. One vial was used for the detection of antibodies against *T. gondii* and *N. caninum* and the second one for the biochemical determination of creatine kinase (CK) and aspartate aminotransferase (AST) serum concentrations. The third vial was used for PCV2 DNA detection by real-time PCR. The samples selected for PCV2 DNA detection, a method description, and the results are presented in the Supplementary Material.

### 2.4. Indirect Immunofluorescence Antibody (IFA) Assay

For the detection of antibodies against *T. gondii* and *N. caninum*, indirect fluorescence antibody test kits using commercially available slides coated with parasite tachyzoites (Fuller Laboratories, Fullerton, CA, USA) and a goat polyclonal fluorescein isothiocyanate (FITC) conjugated anti-pig IgG (Porcine IgG FITC conjugate, VMRD Inc., Pullman, Washington, USA) were used. Nc1 tachyzoites propagated in Vero cells were used for the *N. caninum* slides. After the addition of sera, the slides were incubated for 30 min at 37 °C. The slides were then washed to remove unreacted serum proteins, and fluorescence labelled anti-porcine IgG (conjugate) was added. After incubation for the same time and at the same conditions, the slides were washed again to remove unreacted conjugate.

For the detection of antibodies against *T. gondii* and *N. caninum*, cutoff values of 1:64 and 1:50 were used, respectively. A Nikon Eclipse E-400 fluorescence microscope was used for the observation (objective × 40).

### 2.5. Biochemical Analysis

Serum levels of CK and AST were measured by an automated biochemical analyzer (Advia^®^ 1800 chemistry analyzer, Siemens Healthineers, Erlangen, Germany) using the commercial diagnostic kits,** employing the analytical kinetic methods of N-acetyl cysteine (NAC) activated for CK measurement and the catalytic concentration measurement of AST of the International Federation of Clinical Chemistry.

### 2.6. Data Analysis

A Chi squared test was run to determine the significance of difference in the percentage of seropositivity between vaccinated and unvaccinated pigs, as well as between 1st and 2nd parity sows, using the statistical software MEDCALC 9.2.

The differences in muscle enzyme activity between seropositive and seronegative to *T. gondii* and/or *N. caninum* pigs were determined in three groups of animals: the (T+) group consisted of animals with IgG against only *T. gondii*, the (N+) group consisted of animals with IgG against only *N. caninum,* and the Neg group included seronegative animals for both pathogens. These data were analyzed using the statistical program JASP 14.1. The normality of the data distribution was assessed with the Shapiro–Wilk test. The data for the serum activity of CK and AST were not normally distributed, and Kruskal–Wallis and Mann–Whitney U tests were used to determine the significance of their differences among groups. A value of *p* ≤ 0.05 was considered significant in all comparisons.

## 3. Results

Of a total number of 380 blood samples, 16 were excluded from the study due to hemolysis. Out of the 364 swine serologically examined by the IFA assay, 16 (4.4%) animals were IgG positive against *T. gondii*, 13 (3.5%) animals were IgG positive against *N. caninum*, and 2 (0.5%) animals were IgG positive against both *T. gondii* and *N. caninum*.

**Figure 1:**
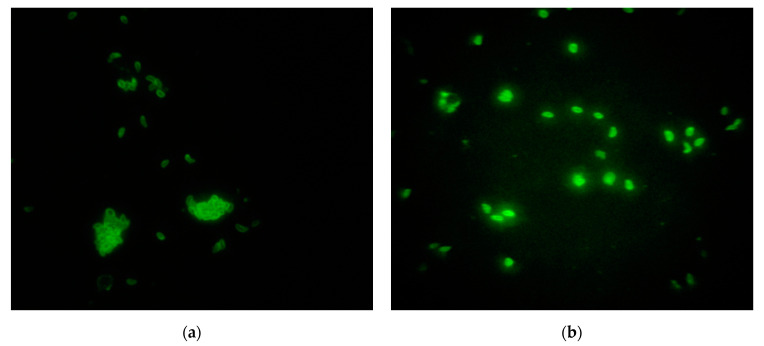
Image of indirect immunofluorescence antibody (IFA) assay observed by a Nikon Eclipse fluorescence microscope (objective ×40). (**a**) *Neospora caninum* positive IgG antibody reaction, serum titer 1:100; (**b**) *Toxoplasma gondii* positive IgG antibody reaction, serum titer 1:128.

Fixed *N. caninum* and *T. gondii* tachyzoites indirectly stained by FITC (IFA) appeared sharp bright green colored with a diffuse or peripheral fluorescence pattern, representing a positive IgG antibody reaction (Figure 1).

The questionnaire revealed that vaccination against PCV2 was not included in the vaccination scheme of 7 out of the 31 farms sampled in the present study. A total number of 45 blood samples was collected from sows of these farms (Table 2). Out of these 45 blood samples, 3 had already been excluded as they belonged to the 16 samples with hemolysis.

Statistical analysis revealed significantly higher percentages of seropositivity against *T. gondii* and/or *N. caninum* in PCV2 unvaccinated compared to vaccinated pigs (Table 3).

No significant difference in seropositivity to *T. gondii* and *N. caninum* was observed between 1st and 2nd parity sows (Table 4).

For the analysis of CK and AST activity, the two animals positive against both *T. gondii* and *N. caninum* have not been included, as groups were formed by seropositive animals to only one pathogen. Therefore, group (T+) consisted of 14 animals, group (N+) consisted of 11 animals, and group (Neg) consisted of 337 animals.

**Figure 2:**
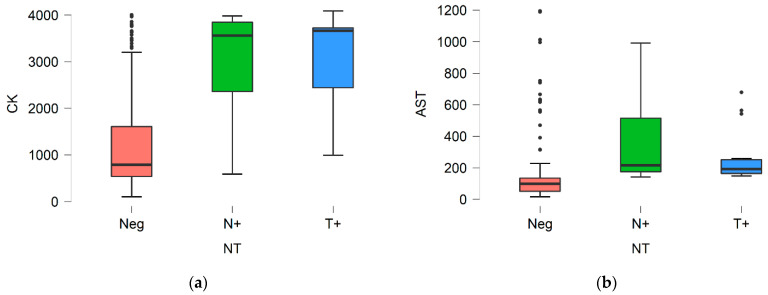
Dot and box and whisker plots of: (**a**) creatine kinase (CK) in pigs seronegative to both *N. caninum* and *T. gondii* (Neg), seropositive to *N. caninum* and seronegative to *T. gondii* (N+), and seropositive to *T. gondii* and seronegative to *N. caninum* (T+); (**b**) aspartate aminotransferase (AST) in pigs seronegative to both *N. caninum* and *T. gondii* (Neg), seropositive to *N. caninum* and seronegative to *T. gondii* (N+), and seropositive to *T. gondii* and seronegative to *N. caninum* (T+).

The serum activities of CK and AST were significantly higher in the T+ and N+ groups compared with the Neg group (*p* < 0.05) (Figure 2). However, no significant difference was detected between the T+ and N+ groups (*p* > 0.05).

**Figure 3:**
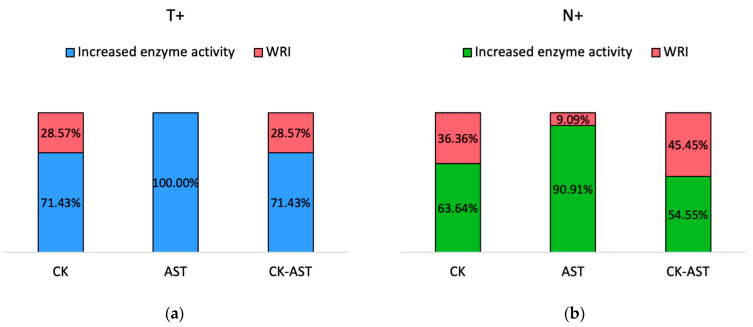
The percentages of sows with increased creatine kinase (CK), aspartate aminotransferase (AST), and both CK-AST serum activities, as well as the percentages of serum enzyme levels within reference interval (WRI), are presented in percentage stacked columns in (**a**) seropositive to *T. gondii* sows (T+) group; (**b**) seropositive to *N. caninum* sows (N+) group.

A percentage of 71.43% of the T+ group and 63.64% of the N+ group presented increased CK activity, while 100% and 90.91% of the groups T+ and N+ presented increased AST activity, respectively (Figure 3). Increased serum levels for both enzymes were detected in 71.43% of the T+ and 54.55% of the N+ group. Both CK and AST enzyme activities were elevated in the two animals that were seropositive against both *T. gondii* and *N. caninum.* Our laboratory reference intervals for CK and AST were 50–3521 U/L and AST 16–142 U/L, respectively.

## 4. Discussion

This study revealed that 4.4% and 3.5% of sows were exposed to *T. gondii* and *N. caninum*, while coexposure was also evidenced in two (0.5%) animals. The farms sampled represent a population of around 11,500 sows, 20% of the total swine population in Greece. The seroprevalence of 4.4% against *T. gondii* is in accordance, and actually very close to, the previous reporting a seroprevalence of 4.3% in swine in Greece [25]. *T. gondii* seroprevalence in Greece is among the lowest in Europe, compared to other European countries—Serbia, Italy, Switzerland, and Romania having the highest percentage of 23–25%—and compared worldwide, as the pooled prevalence in Europe is 15%, while in Asia it is 21%, in Africa 25%, and in South and North America 23% and 25%, respectively [7].

Regarding *N. caninum*, a seroprevalence of 3.5% was detected in the present study, and this is, to the authors’ best knowledge, the first report on swine exposure to *N. caninum* in Greece. Although antibodies against *N. caninum* have already been detected in Greece, with the highest seroprevalence reported in cattle (20.89–21.03%) [19,20], the seroprevalence in swine seems to be lower than in dogs (7.63%) [16], sheep (2.5–16.8%) [17,18], and goats (6.9%) [18] but higher than the seroprevalence in wild boars (1.1%) [21] and hares (0.95%) [22]. At a global level, the seroprevalence of 3.5% found in Greece is in accordance with the seroprevalences of 3.1–3.2% in Brazil [10,11], 0.3–4.9% in China [13], and 3% in Czech Republic [14] but lower than that of 13.49% in Brazil in 2019 and 15.8% in feral swine in the United States [15]. Regarding the percentage of 0.5% of coexposure to *T. gondii* and *N. caninum*, a similar percentage of 1.5% of coexposure was detected in the Czech Republic [14].

Until the first indication of natural *N. caninum* infection in swine in 2004, most information about neosporosis in this species came from experimental studies. In swine experimentally inoculated with *N. caninum* tachyzoites, the gilts seroconverted (IgG) on the 5th–7th day after Nc1 strain inoculation with the highest IgG titers at the 14th–28th days, which remained stable positive during at least the gestation period and, in total, 130 days post inoculation [36]. Similarly, when sows are inoculated with *T. gondii* oocysts, they seroconverted (IgG) about 2–3 weeks post inoculation and remained seropositive for at least 38 weeks [38]. However, it should be noted that in both studies, IgG antibodies could remain even longer, as a considerable drop in their concentration was not evidenced in the last sampling. Therefore, the results of the present study are indicative of exposure to both pathogens, while the time of infection remains unknown. In the present study, sows on the 10th day of lactation were sampled; thus, infection could have occurred at any time but at least 1–3 weeks prior to sampling, given the time post infection needed for IgG antibodies to be detectable in blood [36,38]. As for any serological test, our results must be interpreted in the light of the pathogenesis of these two protozoans, specifically the timing of antibody appearance in blood. Moreover, apart from the analytical sensitivity and specificity of the IFA assay, the subjectivity and potential interobserver variation of microscopy employed need to be considered. However, samples were evaluated by experienced personnel in the present study, and methods were standardized in our previous studies [21,25]. Finally, the IFA assay is a simple and safe alternative method to the gold standard serological dye test for *T. gondii* antibody detection in humans according to OIE [39].

It is noteworthy that seropositivity to *N. caninum* has been significantly associated with concurrent seropositivity to *T. gondii* in HIV-positive humans, since immunocompromised patients are vulnerable to infection with opportunistic pathogens, such as *N. caninum* and *T. gondii* [28]. The presence of antibodies against *N. caninum* has been evidenced in HIV-infected patients with a high seroprevalence of about 38% [28], attributed mainly to the disturbed mucosal immune barrier of the gastrointestinal tract, due to mucosal CD4+ T lymphocyte depletion and functional impairment [40]. These high seropositivity rates to *N. caninum* raise concerns about the role of dogs as companion animals and definitive hosts of the parasite and the effect of *N. caninum* infection in immunocompromised individuals. Relatedly, a concomitant infection with immunosuppressive viruses such as bovine viral diarrhea virus has been evidenced in cattle, contributing to the severity of *N. caninum* infection [41,42].

Regarding the swine immunization status, seropositivity to *T. gondii* and/or *N. caninum* was significantly higher in unvaccinated pigs compared to pigs vaccinated against PCV2. PCV2 targets the immune system, mainly the monocytes–macrophages cell line of lymphoid tissue and lungs, with immunopathological and immunosuppressive effects [43]. However, the mechanisms involved in its pathogenesis are not clearly known. One immunosuppressive mechanism employed by the PCV2, is the inhibition of antigen presentation by the dendritic cells and subsequent activation of T-cells, mediated by endothelial IL-8, the expression of which is increased after PCV2 infection of the vascular epithelial cells. Specifically, IL-8 reduces the adhesion and migration of dendritic cells resulting in maturation impairment, with the immature cells exhibiting a low antigen presentation ability [44]. In the present study, although seropositivity was reversely associated with PCV2 vaccination, this association needs to further be exploited since PCV2 disease was not diagnosed, but it is more likely to occur in unvaccinated rather than in vaccinated pigs. The presence of PCV2 viral DNA in unvaccinated pigs (Supplementary Material) is an additional clue supporting the potential implication of PCV2, considering its abovementioned pathogenesis as an immunosuppressive factor, which, however, needs to be confirmed. Moreover, the higher seropositivity to *T. gondii* and *N. caninum* in non-PCV2-vaccinated pigs could reflect poor farm management and thus increased exposure to pathogens, including *T. gondii* and *N. caninum.*

Infection with *T. gondii* or *N. caninum* may go unnoticed with a high rate of asymptomatic carriers or may cause a variety of clinical signs depending on the host’s immune status [3,45]. Muscle tropism and persistence of the parasite’s bradyzoite encysted stage may occasionally result in chronic infection, which has been reported to cause a nonresolving myositis with extensive muscle damage due to the persistent accumulation of proinflammatory macrophages [46]. In the present study, CK and AST serum activities were significantly higher in seropositive to *T. gondii* or *N. caninum* sows, compared to seronegative ones for both pathogens. Concurrent increased serum levels of CK and AST were evidenced in 71.43% of the T+ and 54.55% of the N+ group, as well as in the two sows found seropositive to both *T. gondii* and *N. caninum.* Increased serum activities for CK and AST were detected in 71.43% and 100% of sows in group T+ and in 63.64% and 90.91% of sows in group N+.

The measurement of the serum enzyme activity of specific enzyme markers of muscle stress or injury, such as CK and AST, is critical for the evaluation of neuromuscular disorders. CK and AST are cytoplasmic and mitochondrial enzymes that leak into bloodstream as a result of injury or loss of cell membrane integrity. CK is expressed in high concentrations in the skeletal muscles, followed by the myocardial muscles, brain, and intestine, while AST is expressed in several tissues and in erythrocytes, with the highest levels in hepatocytes, myocardial, and skeletal muscle cells [47]. Since AST is widely found in many tissues, increased values are a nonspecific indicator for muscle injury and could be either a result of liver damage or hemolysis [48]. However, the possibility of hemolysis interference in the present study is limited, as all hemolyzed specimens were excluded. Although liver implication cannot be excluded, muscle, more than liver, damage seems to be more likely, since elevations in serum AST activity were interpreted along with concurrent elevations in CK activity. Serum AST half-life is approximately 18 h [47] and longer than serum CK half-life, which is short and approximately 5.2 h in pigs [47,49]. This difference in enzyme half-lives is reflected in the number of animals with elevated muscle activity; CK has a shorter half-life and therefore an elevation in its activity is easier to miss, depending on the time elapsed between sampling and muscle injury.

Increased serum levels of muscle enzymes have been detected after extensive muscular activity in humans [50], dogs [51], and pigs [52], and in pathological conditions of muscle injury like myositis, rhabdomyolysis [53], muscular trauma, muscular dystrophy [54], and acute myocardial injury. There are several references about substance administration and increased muscle enzyme levels due to muscle trauma [55]; however, all animals subjected to injections either for drug administration or blood collection within the last 7 days were excluded from the study. Specifically, in pigs, muscle damage, reflected by elevated serum muscle enzyme activities, has been described in several pathological conditions. Myofiber degeneration and necrosis has been reported in mulberry heart disease and muscular dystrophy, which both have been associated with selenium and vitamin E deficiency [56,57]. However, in the present study, the administration of a balanced diet was within the inclusion criteria and the occurrence of such pathological conditions was rather unlikely. The same holds true for iron, gossypol, or ionophore toxicosis as causes of muscle disease that have been excluded in the farm selection process.

Except for nutritional causes and toxicoses, secondary causes, such as infectious agents and porcine stress syndrome (PSS), can lead to myocardial or skeletal muscle cells degeneration and necrosis [57]. PSS has been associated with an autosomal recessive gene, with the genetic predisposed animals being susceptible and presenting exertional stress under natural conditions and suddenly dying [58]. In an experimental study in stress-susceptible pigs, severe myocardial and skeletal muscle necrosis with a pronounced CK elevation were evidenced after exposure to halothane [59], a stimulant used to test PSS predisposition [60]. In the present study, sampling was performed with the minimal stress induction in sows, and all procedures were done to promote the animals’ welfare. However, although stress was minimized as much as possible, it cannot be totally excluded as a partial or overall cause for the increased muscle enzyme levels.

As PCV2 is associated with multisystemic disease, muscle involvement has also been described, including the presence of granulomatous necrotizing myositis of skeletal muscles accompanied by locomotor clinical signs [61] and acute necrotizing or chronic fibrosing myocarditis [62]; however, both studies refer to growing and fattening pigs, with the infected pigs aged 28–120 days old. Elevated muscle enzyme activities could have been a result of trichinellosis. Trichinella spiralis causes structural remodeling and biochemical disturbances in muscle tissue by the larvae invasion in myocytes, growth, and encapsulation [63]. However, trichinellosis in Greece is highly unlikely in commercial farms, since an active surveillance program is carried out in all European countries, according to EC regulation 1375/2015 [64], so cases of trichinellosis are sporadically observed only in wild or free-range pigs [65].

Regarding the only previous reference to AST and neosporosis in pigs, an increase was also observed in accordance with the present study [36]. However, in that case, AST was used along with GGT testing to identify liver and not muscle damage, with the latter enzyme remaining within the reference interval [36], suggestive of muscle, and not liver, involvement.

The presence of antibodies against *T. gondii* and *N. caninum* raises concerns about the source and the factors contributing to the infection or possible reactivation of a latent infection, as indicated by the laboratory-evidenced muscle damage. Among these factors, host immunity status, as indicated in the present study by the highest seropositivity rates for both pathogens in sows unvaccinated against PCV2, seems to play a critical role and needs further elucidation. Moreover, epidemiological studies should be regularly repeated to ascertain the epidemiological status and to define the preventative measures. While *T. gondii* infection, as a global opportunistic zoonotic parasite, has been broadly studied in humans and animals, the effect of *N. caninum* infection in humans remains unclear. Thus, the elucidation of the pathogenicity of *N. caninum* swine infection in further documented immunocompromised and immunocompetent pigs will also serve as a model for human disease.

## 5. Conclusions

In the present study, evidence of the exposure of pigs to *T. gondii* and, for the first time, *N. caninum* in Greece is provided. Moreover, the presence of antibodies was associated with increased muscle enzyme activity, suggestive of muscle injury, especially in sows with potentially altered or suppressed immunity, either due to pregnancy or to lack of vaccination against the immunosuppressive virus PCV2. Although the importance of seroprevalence studies on *T. gondii* is well documented in pigs due to its zoonotic potential, the evidence of *N. caninum* exposure or infection in humans highlights the necessity of similar studies for *N. caninum* in pigs, at least as an animal model of *N. caninum* pathogenesis, especially in immunocompromised animals.

## Figures and Tables

**Table 1:** Number of blood samples collected per farm of different farm size.

Farm Size ^1^	Farms	Blood Samples
50–100	6	30
101–250	10	100
251–500	10	150
>500	5	100

^1^ Number of sows per farm.

**Table 2:** Number of porcine circovirus 2 (PCV2) unvaccinated farms to total number of farms and number of blood samples collected from PCV2 unvaccinated farms to total number of blood samples, per farm size.

Farm Size ^1^	PCV2 Unvaccinated Farms/Total Farms	Blood Samples from Unvaccinated Farms/Total Blood Samples
50–100	5/6	25/30
101–250	2/10	20/100
251–500	0/10	0/150
>500	0/5	0/100

^1^ Number of sows per farm.

**Table 3:** Number and percentage of *T. gondii* and *N. caninum* seropositive pigs per vaccination status.

PCV2 Vaccination Status	Seropositive Pigs *T. gondii* (16) *N. caninum* (13)			
	N	%	N	%
Vaccinated (322)	9	2.79 ^1^	1	0.31 ^1^
Unvaccinated (42)	7	16.67 ^2^	12	28.57 ^2^

^1,2^ Different superscripts in percentages of different rows in the same column denote significant difference, *p* < 0.0001.

**Table 4:** Number and percentage of 1st and 2nd parity sows seropositive against *T. gondii* and *N. caninum*.

Parity	Seropositive Pigs *T. gondii* (16) *N. caninum* (13)			
	N	%	N	%
1st parity (143)	6	4.2	7	4.9
2nd parity (221)	10	4.5	6	2.7

## Data Availability

The data presented in this study are available on request from the corresponding author. The data are not publicly available due to further processing for other studies.

## Supplementary Materials

The following are available online at https://www.mdpi.com/article/10.3390/microorganisms9051097/s1.
