# Supplementary material for: Neospora caninum and/or Toxoplasma gondii Seroprevalence: Vaccination against PCV2 and Muscle Enzyme Activity in Seropositive and Seronegative Pigs
Source: Microorganisms. 2021 May 20;9(5):1097. doi: 10.3390/microorganisms9051097 (PMC8160920; doi:10.3390/microorganisms9051097)
Supplement: Supplementary file 1 [file microorganisms-09-01097-s001.zip › microorganisms-1172347-supplementary.pdf]

Real-time PCR assay

Sample selection for PCV2 DNA detection

Out of the 42 samples from the unvaccinated against PCV2 pigs, a number of 17 samples from pigs seropositive to *T. gondii* and/ or *N. caninum* were selected for PCV2 DNA detection by real-time PCR (Table 1).

Method

All samples were subjected to nucleic acid extraction, using the QIAamp® cador® Pathogen Mini Kit (Qiagen, Hilden, Germany), under the manufacturer's recommendations. The extracts were subsequently tested for the presence of PCV2 genome, by applying the TaqMan probe-based real-time PCR method as previously described [57]. This method targets a 144 bp conserved region of the viral ORF1. The pair of primers used was PCV2-PT-rep6(F) and PCV2-PT-rep149(R) with sequences 5′-CAGCAAGAAGAATGGAAG-3′ and 5′-TTACCCTCCTCGCCAAC-3′, respectively. Reactions were performed on a LightCycler® 2.0 Instrument (Roche Life Science, Vilvoorde, Belgium) and fluorescence data were analyzed using the LightCycler® Software (version 4.1; Roche Life Science).

Results

Out of the 5 unvaccinated sows of T+ group, 4 (80%) were PCR positive to PCV2, while 7 (70%) of the 10 unvaccinated sows of N+ group were PCR positive to PCV2. The two animals seropositive to both pathogens were also PCR positive to PCV2 (Table 1).

Table 1. Number of seropositive sows against *T. gondii* and/or *N. caninum,* number of porcine circovirus 2 (PCV2) vaccinated and unvaccinated seropositive sows and number of PCV2 PCR positive and negative PCV2 unvaccinated sows.

Seropositive sows		PCV2 vaccination status in seropositive sows		PCV2 PCR in unvaccinated sows	
		+	-	+	-
T. gondii (T+)	14	9	5	4	1
N. caninum (N+)	11	1	10	7	3
Both	2	0	2	2	0
Total	27	10	17	13	4

Conclusion

PCV2 circulation is evident in the majority of unvaccinated seropositive to *T. gondii* and/or *N. caninum* pigs.
